# Predicting water quality through daily concentration of dissolved oxygen using improved artificial intelligence

**DOI:** 10.1038/s41598-023-47060-5

**Published:** 2023-11-21

**Authors:** Jiahao Yang

**Affiliations:** University of Cambridge, Cambridge, CB2 1TN UK

**Keywords:** Chemistry, Energy science and technology, Materials science, Mathematics and computing

## Abstract

As an important hydrological parameter, dissolved oxygen (DO) concentration is a well-accepted indicator of water quality. This study deals with introducing and evaluating four novel integrative methods for the prediction of DO. To this end, teaching–learning-based optimization (TLBO), sine cosine algorithm, water cycle algorithm (WCA), and electromagnetic field optimization (EFO) are appointed to train a commonly-used predictive system, namely multi-layer perceptron neural network (MLPNN). The records of a USGS station called Klamath River (Klamath County, Oregon) are used. First, the networks are fed by the data between October 01, 2014, and September 30, 2018. Later, their competency is assessed using the data belonging to the subsequent year (i.e., from October 01, 2018 to September 30, 2019). The reliability of all four models, as well as the superiority of the WCA-MLPNN, was revealed by mean absolute errors (MAEs of 0.9800, 1.1113, 0.9624, and 0.9783) in the training phase. The calculated Pearson correlation coefficients (R_P_s of 0.8785, 0.8587, 0.8762, and 0.8815) plus root mean square errors (RMSEs of 1.2980, 1.4493, 1.3096, and 1.2903) showed that the EFO-MLPNN and TLBO-MLPNN perform slightly better than WCA-MLPNN in the testing phase. Besides, analyzing the complexity and the optimization time pointed out the EFO-MLPNN as the most efficient tool for predicting the DO. In the end, a comparison with relevant previous literature indicated that the suggested models of this study provide accuracy improvement in machine learning-based DO modeling.

## Introduction

### Background

As is known, water quality is a primary indicator of ecosystem health in aquatic communities. For instance, in aquaculture, the quality and growth of aquatic products are highly affected by the quality of water^1^. The concentration of dissolved oxygen (DO) is a well-known measure of water quality, reflecting the balance between the production and consumption of oxygen. Therefore, is an important criterion for water quality management ^2,3^. The variations in DO concentration are functions of several factors, however, major sources of DO are photosynthetic activities, aeration (at structures), and re-aeration (from the atmosphere)^4^.

Measuring the DO is a difficult task due to the effect of various factors like salinity, temperature, oxygen source, etc.^5,6^. Considering this dynamic nature, as well as the challenges in providing DO measurement equipment, developing DO predictive models is of great desire for monitoring water quality. Hence, non-linear methods have received increasing attention for exploring the relationship between the DO and environmental key factors. Water discharge (Q), water temperature (WT), pH, and specific conductance (SC) are among the most important parameters and different combinations of them have been considered in earlier research depending on data availability and environmental conditions.

A very popular provider of these hydrological time series is the US Geological Survey (USGS)^7^. It is a research organization that provides high-quality and publicly available water data for different areas in the US. In general, the provided data are categorized as either (i) approved for publication or (ii) subject to revision. As the names imply, the first group of data has been reliably processed by the relevant staff, while the second group has not received this approval yet. In this work, the approved data of Klamath River Station (station number 11509370) is used. Many studies in the literature on water quality prediction have used USGS data^8,9^, especially for DO prediction of the Klamath River^10,11^.

### Literature review

With recent advances in computational and measurement domains, the world of science has witnessed various developments aiming at facilitating complex problems regarding natural phenomena^12–15^. For instance, remote sensing facilities are among the most applicable tools for monitoring nature e.g., water bodies^16–19^. Hydrology is one of these fields that has been highly befitted by these developments^20–23^. In this sense, the involved subject may extend from precipitation analysis^24,25^ to water quality assessment^26^. Statistical and machine learning methods are two evident examples of suggested models for water quality analysis^27,28^.

In a general sense, prediction/monitoring studies cover a wide range of scientific efforts for various environmental parameters^29–31^. The advent of machine learning has shed new light on this domain as it provides fast, reliable, and inexpensive solutions to complex prediction problems. Recent sophisticated methods like artificial neural network (ANN) and adaptive neuro-fuzzy inference system (ANFIS) have been highly regarded by engineers for DO prediction in different parts of the world^32,33^. Ay and Kisi^34^ investigated the ability of two popular notions of ANNs, namely radial based function (RBF) and multi-layer perceptron neural network (MLPNN) for analyzing the DO concentration. They compared these models with multilinear regression (MLR). It was found that the RBF performs better than the two other models. Liu et al.^35^ successfully employed an attention-based recurrent neural network (RNN) for long-term and short-term prediction of the DO. More research about the effectiveness of the ANNs can be found in^36–38^. Ji et al.^39^ proved the applicability of support vector machine (SVM) for predicting the DO concentration in hypoxic river systems. With reference to a larger than 86% correlation obtained for the testing phase, they used the SVM as a promising approach for this purpose. Huan et al.^40^ demonstrated the high efficiency of the SVM based on the least-squares theory (LSSVM). Shi et al.^41^ successfully applied a clustering-based softplus extreme learning machine (CSELM) for simulating the DO content in aquaculture. It was also shown that this model is more accurate than standard ELM. Kisi et al.^42^ proposed an intelligent model called Bayesian model averaging (BMA), for the DO estimation. They validated the performance of this model by five well-known models including ELM, classification and regression tree (CART), ANN, MLR, and ANFIS. Based on the obtained RMSEs (1.321 for the BMA vs. 1.439, 1.809, 1.504, 1.742, and 1.447 for the ELM, CART, ANN, MLR, and ANFIS, respectively), the superiority of the BMA was clearly derived. Najah et al.^43^ compared the ANFIS with ANN for the DO modeling and found that the results of the ANFIS were more accurate. Olyaie et al.^44^ conducted a comparison among four data-driven models including RBF, linear genetic programming (LGP), MLPNN, and SVM used for the same objective. Referring to the respective coefficient of determinations (R^2^s of 0.8140, 0.9662, 0.9169, and 0.9748), the SVM surpassed other tested models. The feasibility of a so-called deep learning technique “gated recurrent unit” for the DO analysis in a fishery pond was shown by Li et al.^45^. This model also outperformed the RNN and long short-term memory. The applicability of other machine learning models such as evolving fuzzy neural network (EFuNN)^11^, radial basis function neural network (RBFNN) and general regression neural network (GRNN)^46^, long-short term memory (LSTM)^47^, support vector regression (SVR)^48^, dynamic evolving neural-fuzzy inference system (DENFIS)^49^ has been shown and compared in earlier studies. Further comparative studies can be found in earlier literature^50–52^.

Optimization of regular predictive models has been studied by many scholars in recent years^53–55^. Raheli et al.^56^ built an optimized version of the MLP neural network using firefly algorithm for forecasting the DO and biochemical oxygen demand. The performance of the hybrid model was found to be more reliable than the standard MLP. Furthermore, uncertainty analysis revealed an acceptable degree of uncertainty for the ANN. Yaseen et al.^57^ coupled an LSSVM with bat algorithm for approximating the DO. A comparison with conventional machine learning models like multivariate adaptive regression spline (MARS) and M5 tree pointed out a considerably higher accuracy (i.e., 42% and 20% RMSE reduction) for the proposed hybrid. Three optimization techniques of particle swarm optimization (PSO), biogeography‐based optimization, and butterfly optimization algorithm were used by Fadaee et al.^58^ for optimizing the ANFIS applied to the seasonal analysis of the DO. The accuracy of the AFNIS experienced nearly 14, 16, 6, and 13% improvement in the spring, summer, fall, and winter, respectively. Liu et al.^59^ could enhance the accuracy of a least-squares SVR with an improved PSO. A similar application of the PSO was examined by Chen et al.^60^. Bayram et al.^61^ recommended the use of teaching–learning based optimization (TLBO) applied to quadratic regression for stream DO analysis. In a comparative effort by Azma et al.^62^, seven hybrids of MLP with biogeography-based optimization (BBO), sunflower optimization (SFO), atom search optimization (ASO), crow search algorithm (CSA), league championship algorithm (LCA), shuffled frog leaping algorithm (SFLA), and slime mould algorithm (SMA) were tested for DO prediction in the Rock Creek Station (USGS number 01648010) around Washington, USA. Their results showed the higher accuracy of the BBO-based model. Also, an importance assessment of the inputs reflected the largest and lowest importance for the TW and Q, respectively.

### Motivation and contribution

Concerning the promising results obtained by hybrid algorithms, utilizing metaheuristic-empowered models is becoming a research hotspot in a wide range of engineering domains. In order to address the latest developments in this regard, this work employs the TLBO along with sine cosine algorithm (SCA), water cycle algorithm (WCA), and electromagnetic field optimization (EFO) as the training strategies of the MLPNN to predict daily DO using five-year records. The main contribution of these four metaheuristic algorithms to the problem of DO prediction lies in tuning the MLPNN computational variables that are responsible for establishing the relationship between the DO and its influential parameters. Hence, due to the optimization procedure of these algorithms, it can be said that the TLBO, SCA, WCA, and EFO will optimize the non-linear dependency of the DO on water conditions to achieve a reliable prediction for different conditions.

The case study is Klamath River (Oregon and northern California, US) whose initial part suffers from seasonal low water quality. This study also pursues comparing the efficiency of the used algorithms toward achieving a fast, inexpensive, and reliable DO evaluative model. The used models are optimized in terms of hyperparameters, and in the end, practical monolithic formulas are extracted to be used as DO-predictive equations; eliminating the need for running computer-aided programs and GUIs. Hence, the outcomes of this study may provide significant contributions to the early prediction of DO concentration within the Klamath River.

## USGS data and study area

**Figure 1:**
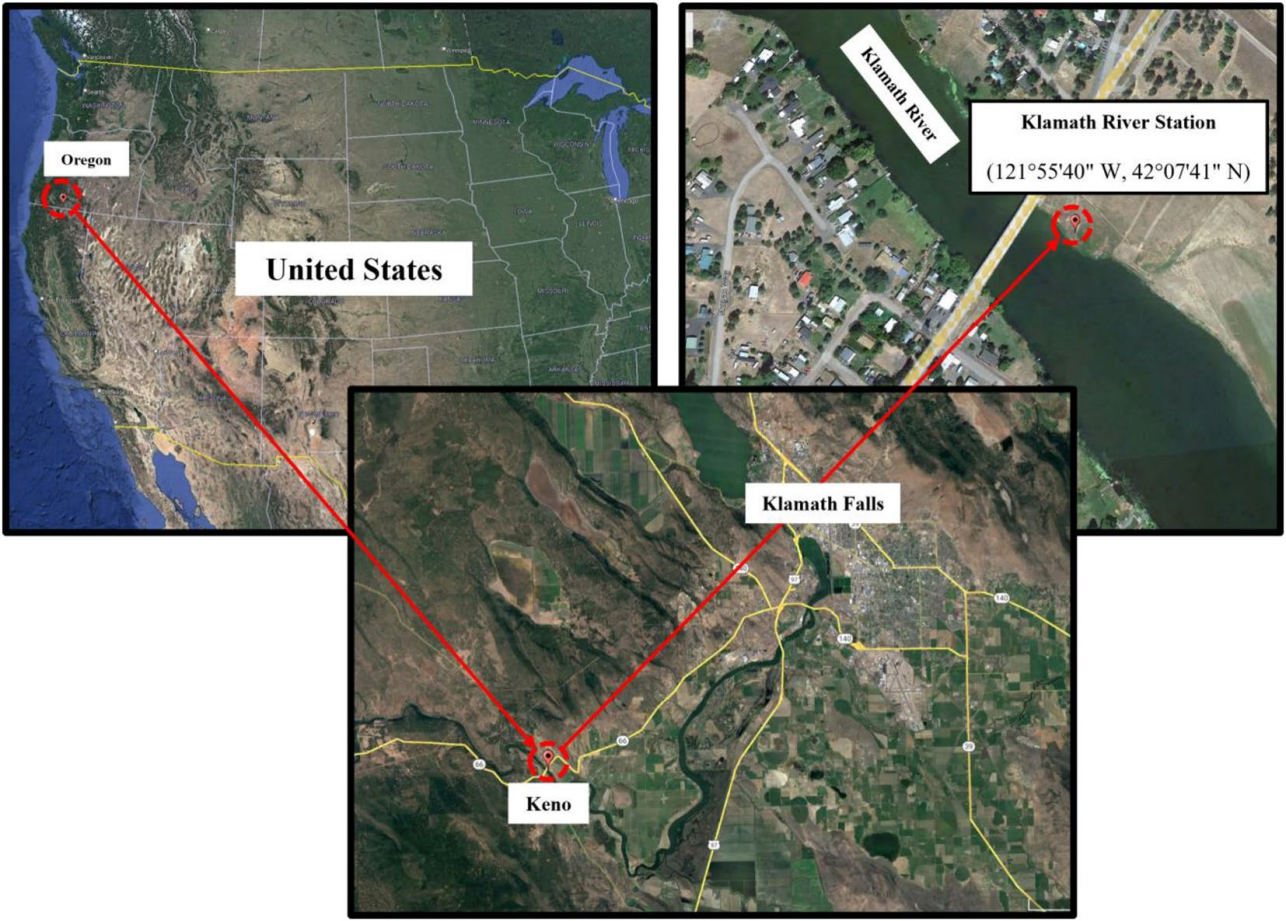
Location of the Klamath River station (images obtained from Google Earth).

Figure 1 shows the location of the study area in Klamath County, Oregon. Flowing through southern Oregon to the Pacific Ocean, the Klamath River has an approximate length of 410 km. It originates from the Link River Dam that is responsible for regulating lake level, controlling downstream flow, and diverting water for hydropower or irrigation purposes. The origin of the Klamath River is a shallow wide reach around Klamath Falls Town (with a rough altitude of 1250 m). The Keno Dam is located around 32 km downstream and controls the river flow. The dominant climate in this area is semi-arid with dry summers and the precipitations mostly occur in the winter (and fall)^49,63^. This initial part of the river is characterized by seasonal low water quality preventing it from hosting aquatics^64^. This issue calls for proper water quality assessment in this area^65^.

**Figure 2:**
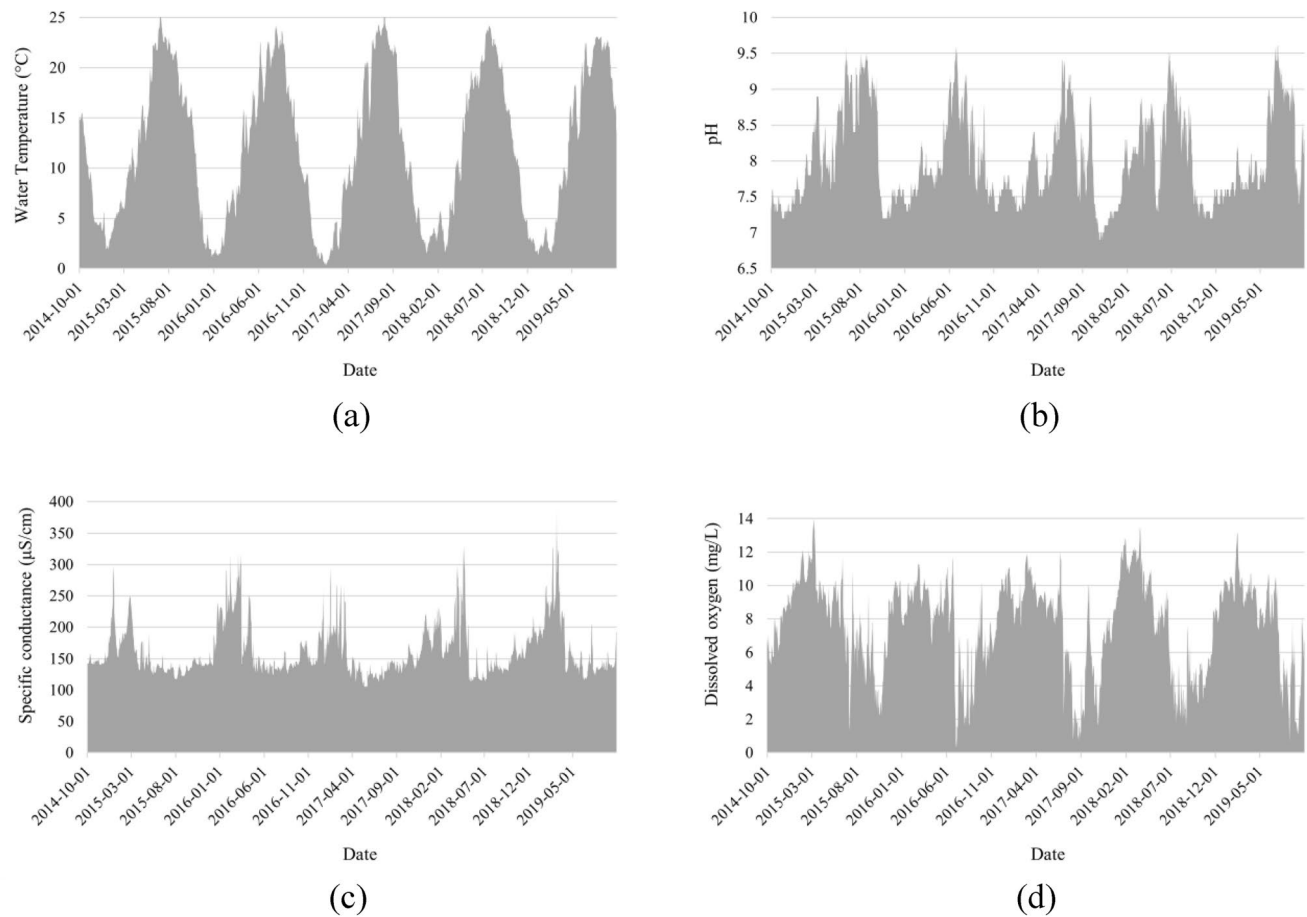
Variations in the DO and independent factors.

The time-series data consisting of WT, pH, SC, and DO records in Klamath River Station operated by USGS (station number 11509370) are downloaded from the USGS water data website (https://waterdata.usgs.gov/nwis). Out of the available data for a five-year period (i.e., 2014–2019), those between October 01, 2014, and September 30, 2018, are considered as training samples for deriving the relationship between the DO and WT, pH, and SC. The trained models are then tested using the data between October 01, 2018, and September 30, 2019, called testing data. Figure 2 depicts the variations in the WT, pH, SC, and DO. Moreover, the training and testing datasets are statistically described in Table 1.

**Table 1:** Descriptive statistics of the used datasets.

Indicator	Train data				Test data			
	WT (°C)	pH	SC (μS/cm)	DO (mg/L)	WT (°C)	pH	SC (μS/cm)	DO (mg/L)
Average	12.20	8.05	160.46	7.74	11.54	7.97	174.20	7.59
Standard deviation	7.38	0.64	39.81	2.78	7.43	0.65	45.80	2.69
Sample variance	54.43	0.41	1584.75	7.72	55.23	0.42	2097.97	7.24
Skewness	0.07	0.48	1.54	-0.54	0.11	0.96	1.49	-0.57
Minimum	0.40	6.90	105.00	0.30	1.40	7.20	116.00	0.60
Maximum	25.70	9.60	332.00	14.00	23.10	9.60	387.00	13.20

## Methodology

**Figure 3:**
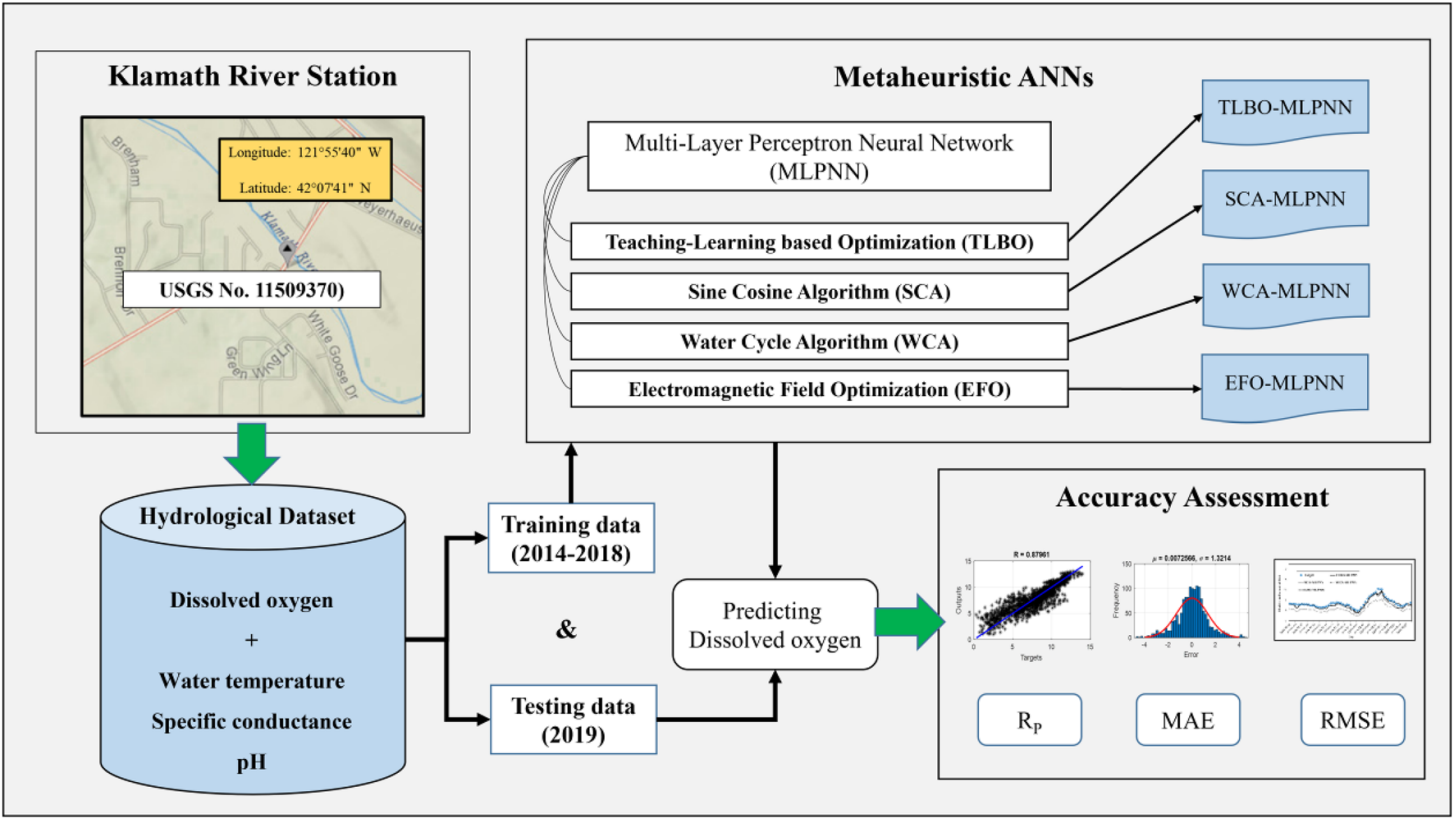
Methodology of this study.

Figure 3 shows the methodological flowchart of the study. After data provision from the Klamath River station, training and testing datasets are created. The models are developed by combining the MLPNN model with four metaheuristic algorithms of TLBO, SCA, WCA, and EFO. These models are trained using the training dataset and they predict the DO for the testing period. In the end, their accuracy is evaluated using error and correlation criteria to rank their performance.

In the following, the description of the models is presented.

### The MLPNN

**Figure 4:**
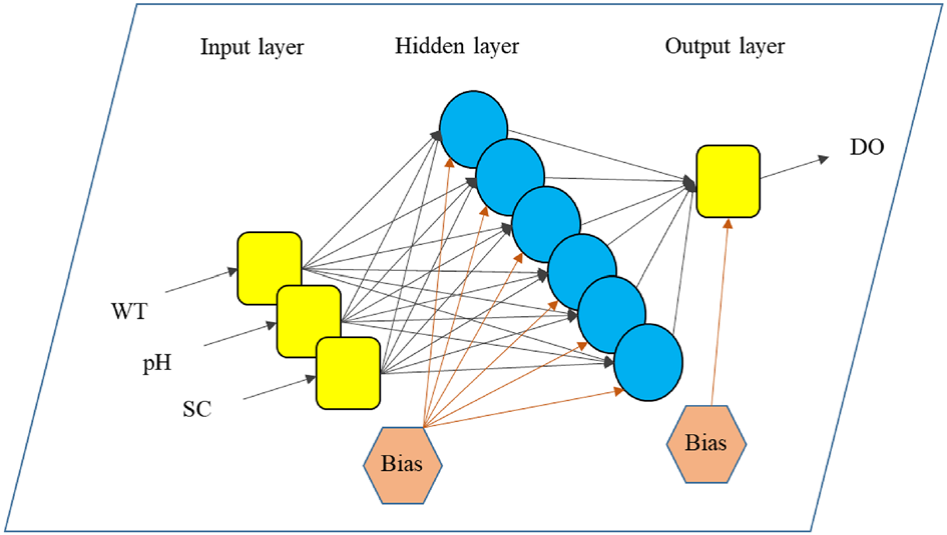
The MLP designed for predicting the DO.

The MLPNN^66,67^ is a broadly used type of ANNs^68^ that is structured on several units lying in three (or more) layers, namely the input layer, hidden layer(s), and output layer. Figure 4 shows the architecture of the MLPNN used in this work. The neurons in an MLPNN are completely connected together. The weights of the network play the role of synapses in a biological neural network.

In each neuron, the input is multiplied by a specific weight factor, and then, added to a bias. The neurons in the hidden layer and output layer can have a linear or non-linear activation function that releases the outcome of the neurons in the last step.

The training mechanism of an MLPNN is described as iteratively adjusting the weights and biases toward a more accurate prediction (e.g., a lower error) for the new network. A common algorithm that is responsible for this process is Levenberg–Marquardt^69^. In this work, this algorithm is replaced with TLBO, SCA, WCA, and EFO.

### Metaheuristic algorithms

The TLBO is a metaheuristic algorithm designed by Rao et al.^70^. It has been widely used for solving various problems^71^. In this algorithm, a class (with the students and their teacher) is simulated so that the teacher influences the learners to reach the most proper harmony. Improving the knowledge of the students takes place in two separate steps conducted by the teacher and students themselves (i.e., teacher phase and learner phase, respectively). In this regard, the potential (i.e., the fitness) of each individual is assessed by exams. In the teacher phase, after calculating the fitness values, the most potent individual is considered a teacher. In the next phase, the learners help together to improve each other's knowledge. Previous studies have detailed mathematical regulations of the TLBO^72,73^.

As a recently developed algorithm, the SCA mimics mathematical rules (i.e., sine/cosine functions). This algorithm was proposed by Mirjalili^74^. After generating a random swarm, the algorithm conducts the optimization over two phases, namely exploration and exploitation. In the first phase, a suitable searching area is found by abruptly mixing the random solution with several others having a large rate of randomness. In the second phase, the random solutions experience changes gradually. Several random values are used in the SCA. Some are considered as the variables of the sine/cosine functions. A random number also plays the role of a criterion for determining the updating equation (i.e., utilizing either sine or cosine function). The SCA has been mathematically described in studies like^75,76^.

Eskandar et al.^77^ developed the WCA by taking the main inspiration from the water cycle running in nature. Assuming that the algorithm commences by raining, the raindrops may finally take the form of a stream, river, and sea-based on their fitness value. In this designation, the sea is the most capable solution provided by the algorithm so far. The rivers also represent an improved version of the streams. These individuals iteratively replace each other to find the most powerful sea. More clearly, once a stream is more promising than a river, they exchange their position. The sea is likewise replaced with a more promising river. In the WCA, the mentioned process is repeated by repeating the rain process. It creates new raindrops and hereby prevents premature optimums. The WCA is detailed in earlier literature^78,79^.

As an electromagnetics-based search scheme, Abedinpourshotorban et al.^80^ proposed the EFO in 2016. Similar to the initial classification executed in the WCA, each agent of the EFO algorithm, known as an electromagnetic particle (EMP), is first grouped in one of the positive, negative, and neutral fields. It is done with respect to the fitness of the proposed EMP. In each iteration, a new EMP is generated and if it brings a larger fitness, it replaces the worst existing EMP. Producing the new EMP begins with taking a member from each field. In the next step, the neutral EMP donates its position (and pole) to the new particle. Based on the fact that EMs with different poles attract each other (and vice versa), the new particle is then affected by positive and negative EMPs. Studies like^81,82^ contain the mathematical details of the explained process.

### Accuracy criteria

For assessing the capability of these models, mean absolute error (MAE) and root mean square error (RMSE) indices are employed to report the prediction error. Equations 1 and 2 describe the error calculation using the MAE and RMSE. Besides, Pearson correlation coefficient (R_P_) is used to measure the correlation of the results. Equation 3 formulates the R_P_ index. Another criterion called Nash–Sutcliffe efficiency (NSE) coefficient is also expressed by Eq. 4.

1 $$ MAE = \frac{1}{Q}\sum\limits_{i = 1}^{Q} {\left| {DO_{{i_{expected} }} - DO_{{i_{predicted} }} } \right|} $$

2 $$ RMSE = \sqrt {\frac{1}{Q}\sum\limits_{i = 1}^{Q} {\left[ {\left( {DO_{{i_{expected} }} - DO_{{i_{predicted} }} } \right)} \right]}^{2} } $$

3 $$ R_{{P_{{}} }} = \frac{{\sum\limits_{i = 1}^{Q} {\left( {DO_{{i_{predicted} }} - \overline{DO}_{predicted} } \right)\left( {DO_{{i_{\exp ected} }} - \overline{DO}_{expected} } \right)} }}{{\sqrt {\sum\limits_{i = 1}^{Q} {\left( {DO_{{i_{predicted} }} - \overline{DO}_{predicted} } \right)^{2} } } \sqrt {\sum\limits_{i = 1}^{Q} {\left( {DO_{{i_{expected} }} - \overline{DO}_{expected} } \right)^{2} } } }} $$

4 $$ NSE = 1 - \frac{{\sum\limits_{i = 1}^{Q} {\left( {DO_{{i_{expected} }} - DO_{{i_{predicted} }} } \right)^{2} } }}{{\sum\limits_{i = 1}^{Q} {\left( {DO_{{i_{expected} }} - \overline{DO}_{expected} } \right)^{2} } }} $$

where ${DO}_{{i}_{predicted}}$ and ${DO}_{{i}_{expected}}$ stand for the modeled and measured DOs, respectively (with respective means of ${\overline{DO} }_{predicted}$ and ${\overline{DO} }_{expected}$). Moreover, *Q* signifies the number of processed samples which equals 1430 and 352 for the training and testing data, respectively.

## Results and discussion

Once again, this paper offers four novel models for DO prediction. The models are composed of an MLP neural network as the core and the TLBO, SCA, WCA, and EFO as the training algorithms. All models are developed and implemented in the MATLAB 2017 environment.

### Optimization and training

Proper training of the MLP is dependent on the strategy employed by the algorithm appointed for this task (as described in previous sections for the TLBO, SCA, WCA, and EFO). In this section, this characteristic is discussed in the format of the hybridization results of the MLP.

An MLPNN is considered the basis of the hybrid models. As per Section “The MLPNN”, this model has three layers. The input layer receives the data and has 3 neurons, one for each of WT, pH, and SC. The output layer has one neuron for releasing the final prediction (i.e., DO). However, the hidden layer can have various numbers of neurons. In this study, a trial-and-error effort was carried out to determine the most proper number. Ten models were tested with 1, 2, …, and 10 neurons in the hidden layer and it was observed that 6 gives the best performance. Hence, the final model is structured as 3 × 6 × 1. With the same logic, the activation function of the output and hidden neurons is respectively selected Pureline (*x* = *y*) and Tansig (described in Section “Formula presentation”) ^83^.

**Figure 5:**
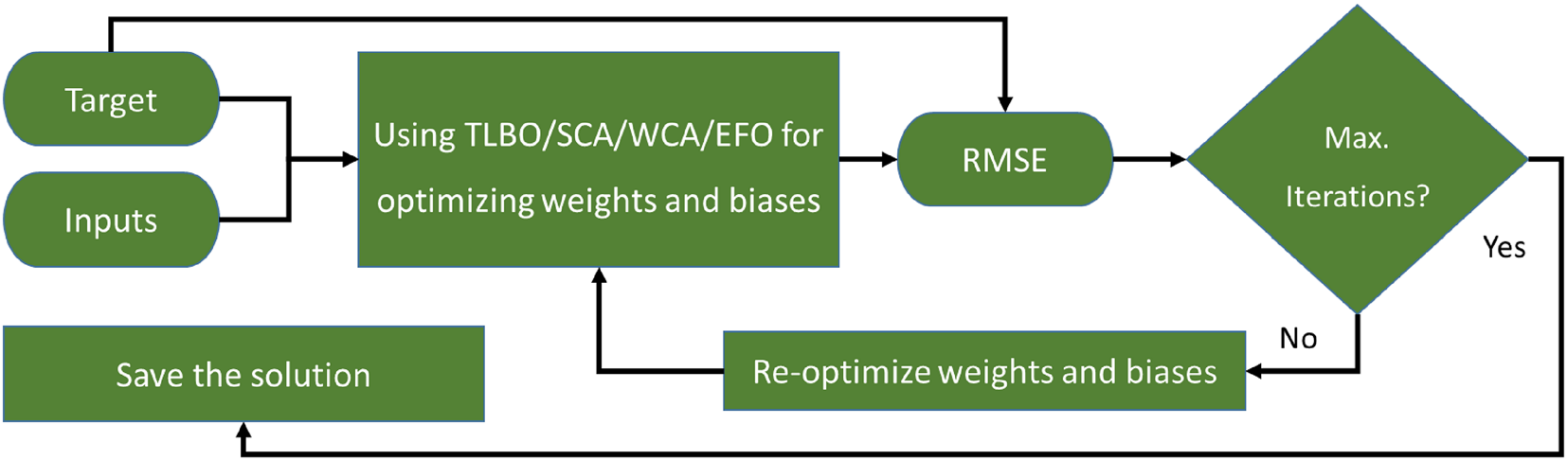
Optimization flowchart of the models.

Next, the training dataset was exposed to the selected MLPNN network. The relationship between the DO and water conditions is established by means of weights and biases within the MLPNN (Fig. 4). In this study, the role of tuning theses weighst and biases is assigned to the named metaheuristic algorithms. For this purpose, the MLPNN configuration is first transformed in the form of mathematical equations with adjustable weights and biases (The equations will be shown in Section “Formula presentation”). Training the MLPNN using metaheuristic algorithms is an iterative effort. Hereupon, the RMSE between the modeled and measured DOs is introduced as the objective function of the TLBO, SCA, WCA, and EFO. This function is used to monitor the optimization benhavior of the algorithms. Since RMSE is an error indicator, the algorithms aim to minimize it over time to improve the quality of the weights and biases. Designating the appropriate number of iterations is another important step. By analyzing the convergence behavior of the algorithms, as well as referring to previous similar studies, 1000 iterations were determined for the TLBO, SCA, and WCA, while the EFO was implemented with 30,000 iterations. The final solution is used to constrcuct the optimized MLPNN. Figure 5 illustrates the optimization flowchart.

**Figure 6:**
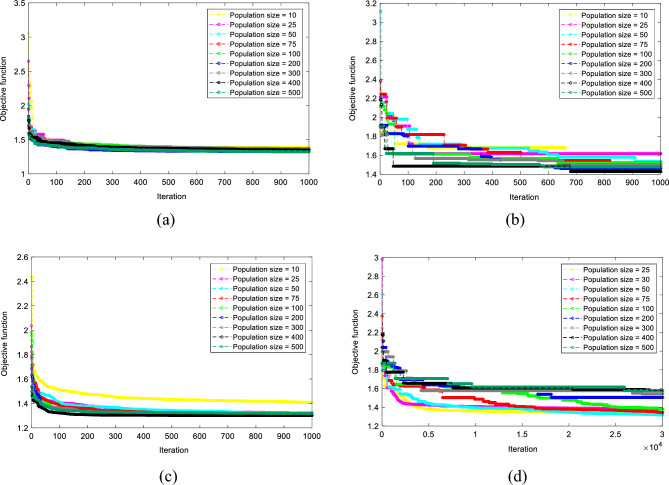
Optimization curves of the (**a**) TLBO-MLPNN, (**b**) SCA-MLPNN, (**c**) WCA-MLPNN, and (**d**) EFO-MLPNN.

Furthermore, each algorithm was implemented with nine swarm sizes (*N*_*SW*_s) to achieve the best model configuration. These tested *N*_*SW*_s were 10, 25, 50, 75, 100, 200, 300, 400, and 500 for the TLBO, SCA, WCA, while 25, 30, 50, 75, 100, 200, 300, 400, and 500 for the EFO^84^. Collecting the obtained objective functions (i.e., the RMSEs) led to creating a convergence curve for each tested *N*_*SW*_s. Figure 6 depicts the convergence curves of the TLBO-MLPNN, SCA-MLPNN, WCA-MLPNN, and EFO-MLPNN.

As is seen, each algorithm has a different method for training the MLPNN. According to the above charts, the TLBO-MLPNN, SCA-MLPNN, WCA-MLPNN, and EFO-MLPNN with respective *N*_*SW*_s of 500, 400, 400, and 50, attained the lowest RMSEs. It means that for each model, the MLPNNs trained by these configurations acquired more promising weights and biases compared to eight other *N*_*SW*_s. Table 2 collects the final parameters of each model.

**Table 2:** Parameters of the used algorithms.

Parameter	TLBO-MLPNN	SCA-MLPNN	WCA-MLPNN	EFO-MLPNN
No. of layers	3			
No. of (input, hidden, output) neurons	(3, 6, 1)			
Activation function of (input, hidden, output) neurons	(-, Tansig, Purelin)			
N_SW_	500	400	400	50
Iterations	1000	1000	1000	30,000

### Training and testing results

**Figure 7:**
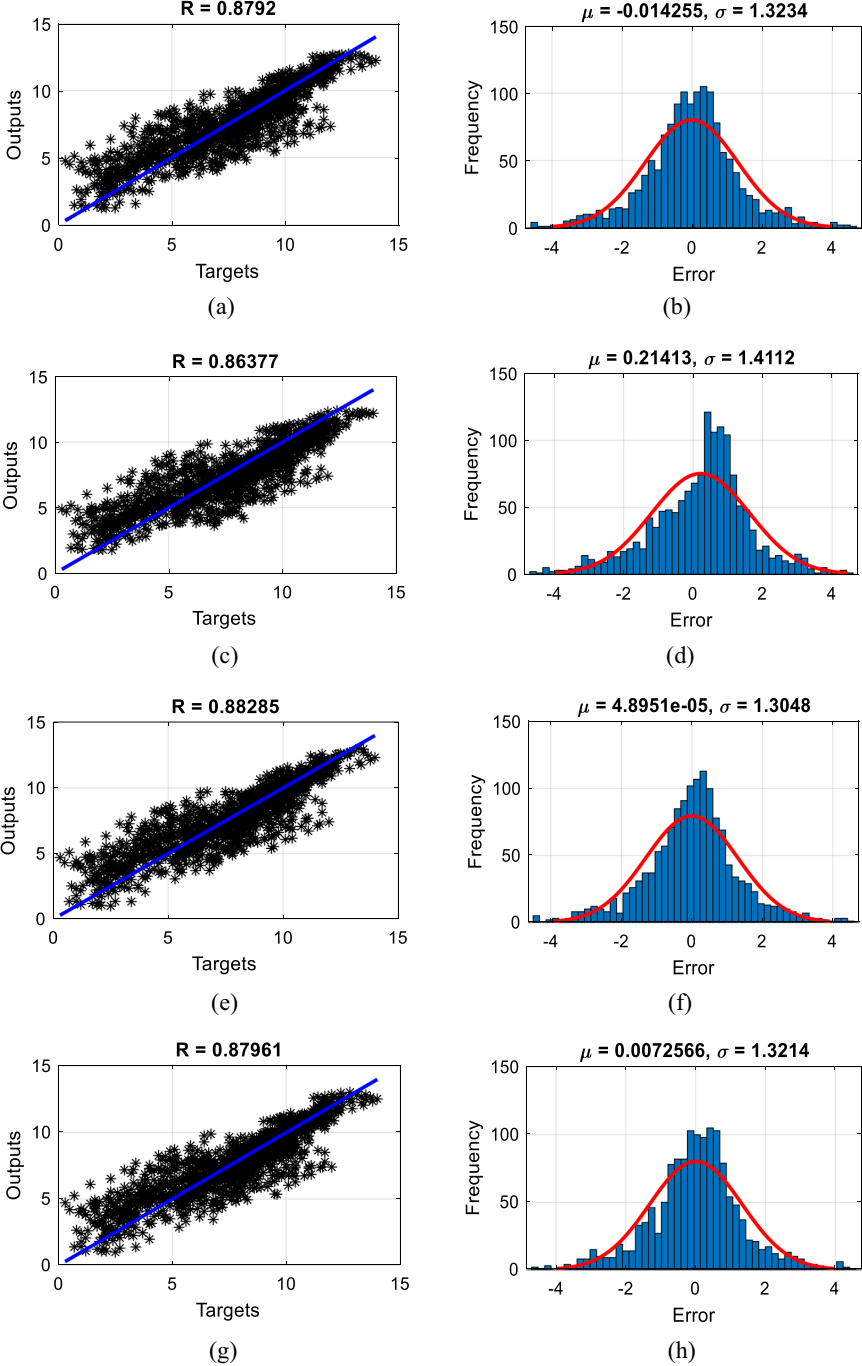
The scatterplot and histogram of the errors plotted for the training data of (**a** and **b**) TLBO-MLPNN, (**c** and **d**) SCA-MLPNN, (**e** and **f**) WCA-MLPNN, and (**g** and **h**) EFO-MLPNN.

The RMSE of the recognized elite models (i.e., the TLBO-MLPNN, SCA-MLPNN, WCA-MLPNN, and EFO-MLPNN with the *N*_*SW*_s of 500, 400, 400, and 50) was 1.3231, 1.4269, 1.3043, and 1.3210, respectively. These values plus the MAEs of 0.9800, 1.1113, 0.9624, and 0.9783, and the NSEs of 0.7730, 0.7359, 0.7794, and 0.7737 indicate that the MLP has been suitably trained by the proposed algorithms. In order to graphically assess the quality of the results, Fig. 7a,c,e, and g are generated to show the agreement between the modeled and measured DOs. The calculated R_P_s (i.e., 0.8792, 0.8637, 0.8828, and 0.8796) demonstrate a large degree of agreement for all used models. Moreover, the outcome of ${DO}_{{i}_{expected }}- {DO}_{{i}_{predicted}}$ is referred to as “error” for every sample, and the frequency of these values is illustrated in Fig. 7b,d,f, and h. These charts show larger frequencies for the error values close to 0; meaning that accurately predicted DOs outnumber those with considerable errors.

**Figure 8:**
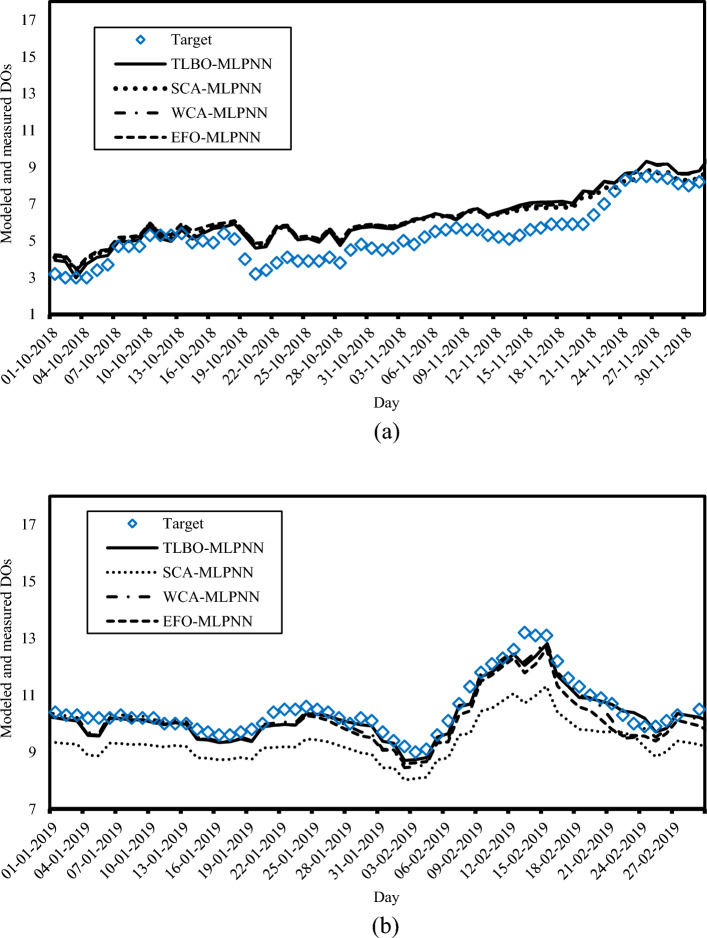
The real and predicted DO patterns for (**a**) October 01, 2018 to December 01, 2018 and (**b**) January 01, 2019 to March 01, 2019.

Evaluating the testing accuracies revealed the high competency of all used models in predicting the DO for new values of WT, pH, and SC. In other words, the models could successfully generalize the DO pattern captured by exploring the data belonging to 2014–2018 to the data of the fifth year. For example, Fig. 8 shows the modeled and measured DOs for two different periods including (a) October 01, 2018 to December 01, 2018 and (b) January 01, 2019 to March 01, 2019. It can be seen that, for the first period, the upward DO patterns have been well-followed by all four models. Also, the models have shown high sensitivity to the fluctuations in the DO pattern for the second period.

**Figure 9:**
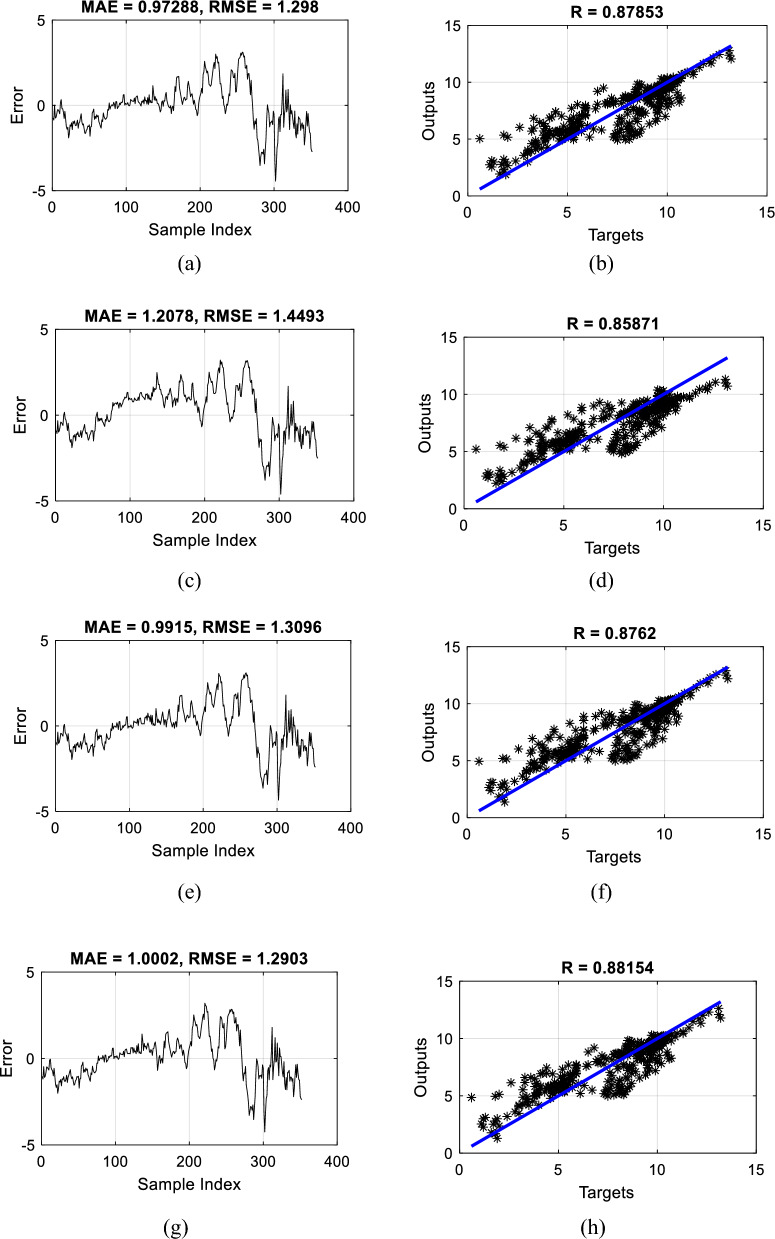
The error line and scatterplot plotted for the testing data of (**a** and **b**) TLBO-MLPNN, (**c** and **d**) SCA-MLPNN, (**e** and **f**) WCA-MLPNN, and (**g** and **h**) EFO-MLPNN.

Figure 9a,c,e, and g show the errors obtained for the testing data. The RMSE and MAE of the TLBO-MLPNN, SCA-MLPNN, WCA-MLPNN, and EFO-MLPNN were 1.2980 and 0.9728, 1.4493 and 1.2078, 1.3096 and 0.9915, and 1.2903 and 1.0002, respectively. These values, along with the NSEs of 0.7668, 0.7092, 0.7626, and 0.7695, imply that the models have predicted unseen DOs with a tolerable level of error. Moreover, Fig. 9b,d,f, and h present the corresponding scatterplots illustrating the correlation between the modeled and measured DOs in the testing phase. Based on the R_p_ values of 0.8785, 0.8587, 0.8762, and 0.8815, a very satisfying correlation can be seen for all used models.

### Efficiency comparison and discussion

To compare the efficiency of the employed models, the most accurate model is first determined by comparing the obtained accuracy indicators, then, a comparison between the optimization time is carried out. Table 3 collects all calculated accuracy criteria in this study.

**Table 3:** Obtained accuracy indices.

Hybrid model	Network results							
	Training phase				Testing phase			
	RMSE	MAE	R_P_	NSE	RMSE	MAE	R_P_	NSE
TLBO-MLPNN	1.3231	0.9800	0.8792	0.7730	1.2980	0.9728	0.8785	0.7668
SCA-MLPNN	1.4269	1.1113	0.8637	0.7359	1.4493	1.2078	0.8587	0.7092
WCA-MLPNN	1.3043	0.9624	0.8828	0.7794	1.3096	0.9915	0.8762	0.7626
EFO-MLPNN	1.3210	0.9783	0.8796	0.7737	1.2903	1.0002	0.8815	0.7695

In terms of all all accuracy criteria (i.e., RMSE, MAE, R_P_, and NSE), the WCA-MLPNN emerged as the most reliable model in the training phase. In other words, the WCA presented the highest quality training of the MLP followed by the EFO, TLBO, and SCA. However, the results of the testing data need more discussion. In this phase, while the EFO-MLPNN achieved the smallest RMSE (1.2903), the largest R_P_ (0.8815), and the largest NSE (0.7695) at the same time, the smallest MAE (0.9728) was obtained for the TLBO-MLPNN. About the SCA-based ensemble, it was shown that this model yields the poorest predictions in both phases.

**Figure 10:**
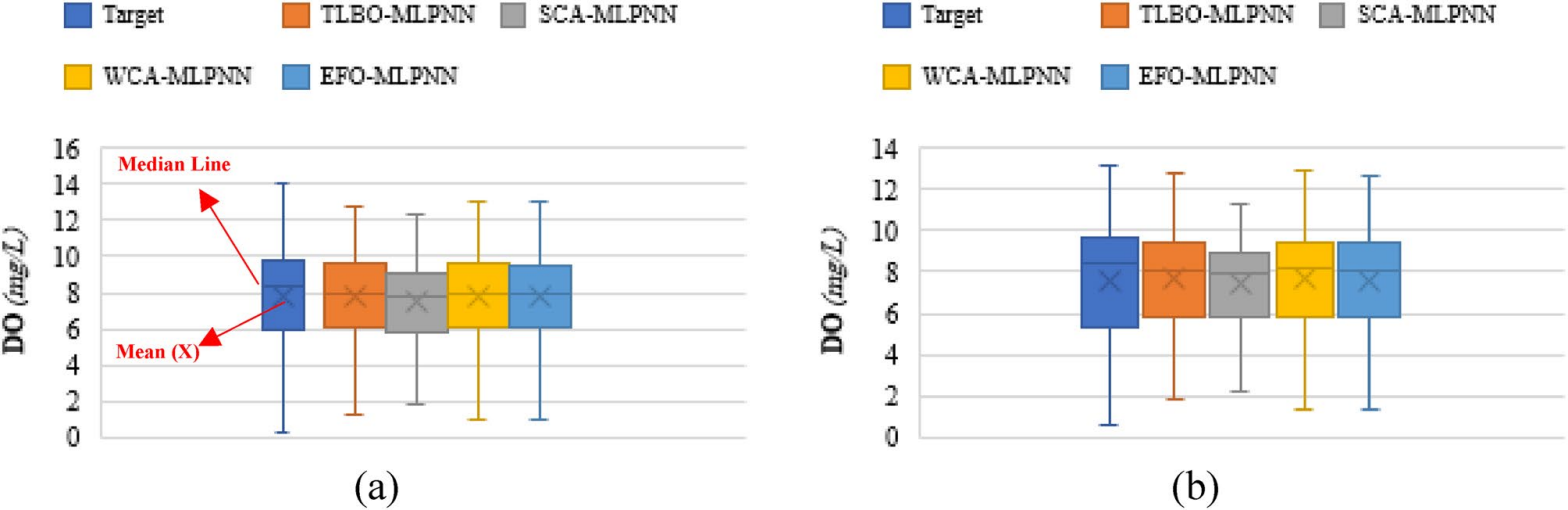
Boxplots of the models for comparison.

**Figure 11:**
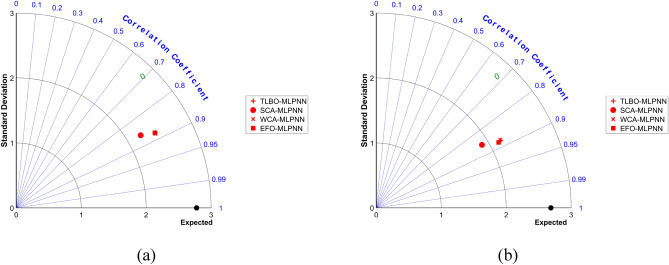
Taylor diagram of the models for comparison.

Additionally, Figs. 10 and 11 are also produced to compare the accuracy of the models in the form of boxplot and Taylor Diagram, respectively. The results of these two figures are consistent with the above comparison. They indicate the high accordance between the models’ outputs and target DOs, and also, they reflect the higher accuracy of the WCA-MLPNN, EFO-MLPNN, and TLBO-MLPNN, compared to the SCA-MLPNN.

In comparison with some previous literature, it can be said that our models have attained a higher accuracy of DO prediction. For instance, in the study by Yang et al.^85^, three metaheuristic algorithms, namely multi-verse optimizer (MVO), shuffled complex evolution (SCE), and black hole algorithm (BHA) were combined with an MLPNN and the models were applied to the same case study (Klamath River Station). The best training performance was achieved by the MLP-MVO (with respective RMSE, MAE, and R_P_ of 1.3148, 0.9687, and 0.8808), while the best testing performance was achieved by the MLP-SCE (with respective RMSE, MAE, and R_P_ of 1.3085, 1.0122, and 0.8775). As per Table 3, it can be inferred that the WCA-MLPNN suggested in this study provides better training results. Also, as far as the testing results are concerned, both WCA-MLPNN and TLBO-MLPNN outperformed all models tested by Yang et al.^85^. In another study by Kisi et al.^42^, an ensemble model called BMA was suggested for the same case study, and it achieved training and testing RMSEs of 1.334 and 1.321, respectively (See Table 5 of the cited paper). These error values are higher than the RMSEs of the TLBO-MLPNN, WCA-MLPNN, and EFO-MLPNN in this study. Consequently, these model outperform benchmark conventional models that were tested by Kisi et al.^42^ (i.e., ELM, CART, ANN, MLR, and ANFIS). With the same logic, the superiority of the suggested hybrid models over some conventional models employed in the previous studies^49,65^ for different stations on the Klamath River can be inferred. Altogether, these comparisons indicate that this study has achieved considerable improvements in the field of DO prediction.

Table 4 denotes the times elapsed for optimizing the MLP by each algorithm. According to this table, the EFO-MLPNN, despite requiring a greater number of iterations (i.e., 30,000 for the EFO vs. 1000 for the TLBO, SCA, and WCA), accomplishes the optimization in a considerably shorter time. In this relation, the times for the TLBO, SCA, and WCA range in [181.3, 12,649.6] *s*, [88.7, 6095.2] *s*, and [83.2, 4804.0] *s*, while those of the EFO were bounded between 277.2 and 296.0 s. Another difference between the EFO and other proposed algorithms is related to two initial *N*_*SW*_s. Since *N*_*SW*_ of 10 was not a viable value for implementing the EFO, two values of 25 and 30 are alternatively considered.

**Table 4:** The time taken for performing the optimum MLP training (In seconds).

Model	No. of iterations	*N*_*SW*_								
		10	25	50	75	100	200	300	400	500
TLBO-MLPNN	1000	181.3	464.2	911.1	1328.0	1835.1	4345.0	8913.5	9533.0	12,649.6
SCA-MLPNN	1000	88.7	220.4	439.2	664.4	884.3	1886.8	2727.2	5295.7	6095.2
WCA-MLPNN	1000	83.2	210.6	448.7	682.1	822.0	1659.4	2504.2	4733.0	4804.0

		*N*_*SW*_								
		25	30	50	75	100	200	300	400	500
EFO-MLPNN	30,000	277.2	287.1	292.6	292.7	288.5	296.0	286.9	291.3	291.2

Based on the above discussion, the TLBO, WCA, and EFO showed higher capability compared to the SCA. Examining the time of the selected configurations of the TLBO-MLPNN, SCA-MLPNN, WCA-MLPNN, and EFO-MLPNN (i.e., 12,649.6, 5295.7, 4733.0, and 292.6 s for the *N*_*SW*_s of 500, 400, 400, and 50, respectively) shows that the WCA needs around 37% of the TLBO’s time to train the MLP. The EFO, however, provides the fastest training.

Apart from comparisons, the successful prediction carried out by all four hybrid models represents the compatibility of the MLPNN model with metaheuristic science for creating predictive ensembles. The used optimizer algorithms could nicely optimize the relationship between the DO and water conditions (i.e., WT, pH, and SC) in the Klamath River Station. The basic model was a 3 × 6 × 1 MLPNN containing 24 weights and 7 biases (Fig. 4). Therefore, each algorithm provided a solution composed of 31 variables in each iteration. Considering the number of tested* N*_*SW*_s and iterations for each algorithm (i.e., 30,000 iterations of the EFO and 1000 iterations of the WCA, SCA, and TLBO all with nine *N*_*SW*_s), it can be said that the outstanding solution (belonging to the EFO algorithm) has been excerpted among a large number of candidates (= 1 × 30,000 × 9 + 3 × 1000 × 9).

However, concerning the limitations of this work in terms of data and methodology, potential ideas can be raised for future studies. First, it is suggested to update the applied models with the most recent hydrological data, as well as the records of other water quality stations, in order to enhance the generalizability of the models. Moreover, further metaheuristic algorithms can be tested in combination with different basic models such as ANFIS and SVM to conduct comparative studies.

### Formula presentation

The higher efficiency of the WCA and EFO (in terms of both time and accuracy) was derived in the previous section. Hereupon, the MLPNNs constructed by the optimal responses of these two algorithms are mathematically presented in this section to give two formulas for predicting the DO. Referring to Fig. 4, the calculations of the output neuron in the WCA-MLPNN and EFO-MLPNN is expressed by Eqs. 5 and 6, respectively.

5 $$ \begin{aligned} DO_{WCA - MLPNN } & = \, 0.395328 \times O_{HN1 } + 0.193182 + O_{HN2 } - 0.419852 \times O_{HN3 } + 0.108298 \times O_{HN4 } \\ & \quad +\, 0.686191 \times O_{HN5 } + 0.801148 \times O_{HN6 } + 0.340617 \\ \end{aligned} $$

6 $$ \begin{aligned} DO_{EFO - MLPNN } & = 0.033882 \times {{O}_{HN1}}^{\prime} - 0.737699 \times {{O}_{HN2}}^{\prime} - 0.028107 \times {{O}_{HN3}}^{\prime} - 0.700302 \\ & \quad \times {{O}_{HN4}}^{\prime} + 0.955481 \times {{O}_{HN5}}^{\prime} - 0.757153 \times {{O}_{HN6}}^{\prime} + 0.935491 \\ \end{aligned} $$

In the above relationships, ${O}_{HNi}$ and ${{O}_{HNi}}^{\prime}$ represent the outcome of the *i*^*th*^ hidden neuron in the WCA-MLPNN and EFO-MLPNN, respectively. Given *Tansig (x)* = $\frac{2}{1+ {e}^{-2x}}$ *– 1* as the activation function of the hidden neurons, ${O}_{HNi}$ and ${{O}_{HNi}}^{\prime}$ are calculated by the below equations. As is seen, these two parameters are calculated from the inputs of the study, i.e., (WT, pH, and SC).

7 $$ \left[ {\begin{array}{*{20}c} {O_{HN1 } } \\ {O_{HN2 } } \\ {O_{HN3 } } \\ {O_{HN4 } } \\ {O_{HN5 } } \\ {O_{HN6 } } \\ \end{array} } \right] = Tansig\left( {\left( {\left[ {\begin{array}{*{20}c} { - 1.818573} & {1.750088} & { - 0.319002} \\ {0.974577} & {0.397608} & { - 2.316006} \\ { - 1.722125} & { - 1.012571} & {1.575044} \\ {0.000789} & { - 2.532009} & { - 0.246384} \\ { - 1.288887} & { - 1.724770} & {1.354887} \\ {0.735724} & { - 2.250890} & {0.929506} \\ \end{array} } \right] \left[ {\begin{array}{*{20}c} {WT} \\ {pH} \\ {SC} \\ \end{array} } \right] } \right) + \left[ {\begin{array}{*{20}c} {2.543969} \\ { - 1.526381} \\ {0.508794} \\ {0.508794} \\ { - 1.526381} \\ {2.543969} \\ \end{array} } \right]} \right) $$

8 $$ \left[ {\begin{array}{*{20}c} {O_{HN1}{\prime} } \\ {O_{HN2}{\prime} } \\ {O_{HN3}{\prime} } \\ {O_{HN4}{\prime} } \\ {O_{HN5}{\prime} } \\ {O_{HN6}{\prime} } \\ \end{array} } \right] = Tansig\left( {\left( {\left[ {\begin{array}{*{20}c} {1.323143} & { - 2.172674} & { - 0.023590} \\ {1.002364} & {0.785601} & {2.202243} \\ {1.705369} & { - 1.245099} & { - 1.418881} \\ { - 0.033210} & { - 1.681758} & {1.908498} \\ {1.023548} & { - 0.887137} & { - 2.153396} \\ {0.325776} & { - 1.818692} & { - 1.748715} \\ \end{array} } \right] \left[ {\begin{array}{*{20}c} {WT} \\ {pH} \\ {SC} \\ \end{array} } \right] } \right) + \left[ {\begin{array}{*{20}c} { - 2.543969} \\ { - 1.526381} \\ { - 0.508794} \\ { - 0.508794} \\ {1.526381} \\ {2.543969} \\ \end{array} } \right]} \right) $$

More clearly, the integration of Eqs. (5 and 7) results in the WCA-MLPNN formula, while the integration of Eqs. (6 and 8) results in the EFO-MLPNN formula. Given the excellent accuracy of these two models and their superiority over some previous models in the literature, either of these two formulas can be used for practical estimations of the DO, especially for solving the water quality issue within the Klamath River.

## Conclusions

Four stochastic search strategies, namely teaching–learning-based optimization, sine cosine algorithm, water cycle algorithm, and electromagnetic field optimization were used to train an artificial neural network for predicting the dissolved oxygen of the Klamath River, Oregon, US. After designating the appropriate parameters for each algorithm, accuracy indices showed that all four methods can properly train the MLP to grasp a reliable understanding of the DO behavior. Due to the same reason, the models could reliably predict the DO for new environmental conditions. The hybrid models were compared in terms of accuracy, complexity, and computation time to detect the most efficient predictor. During the training process, it was deduced that although the EFO algorithm required 30 times more iterations, it accomplished this process far faster than three other algorithms. It also presented the most accurate results (in terms of the RMSE, R_P_, and NSE) in the testing phase. Another advantage of this model was hiring a smaller number of search agents to find the optimal response. After that, the WCA-MLPNN emerged as the second-efficient model. Therefore, two DO predictive, based on the weights and biases tuned by the WCA and EFO were proposed in the last part of this research. Moreover, it was shown that the outstanding models of this study outperform several hybrid and conventional models from previous studies, indicating an improvement in practical DO predictions. It would also help in better solving the problem of poor water quality in the studied area.

## Data Availability

All data analysed during this study was (can be) freely downloaded from the USGS water data website (https://waterdata.usgs.gov/nwis). Also, the code of the used algorithms is available upon reasonable request from the author.
